# The National Pension Fund for Nurses

**Published:** 1889-05-25

**Authors:** 


### The National Pension Fund for Nurses.

The committees of many institutions have so frequently of late desired to have a scheme for discussion that the following proposed for the governors of St. Mary's Hospital, and adopted by them on the 17th inst., may prove of interest to many:

The National Pension Fund for Nurses has been established to afford advantages to nurses to secure annuities in old age.

The first questions that arise are:

1. Is the Pension Fund financially sound?

2. Are its terms as reasonable as those obtainable elsewhere?

1. As to its financial soundness, it need simply be said that Lord Rothschild, Henry H. Gibbs, Esq., E. A. Hambro, Esq., and Junius S. Morgan, Esq., have each deposited £5,000, or £20,000 in all, with the Accountant-General in Chancery as security for the policyholders.

2. With respect to the terms offered, I have carefully compared the premiums set forth in the published tables of the fund with those charged in the case of Government annuities, and I find them to be lower.*

Special Advantages.—The National Pension Fund is to be administered on the mutual system, and all the profits accruing, whether from invested moneys, savings in working expenses, or from any other source, will be devoted to increasing the pensions.

In addition to this, a bonus fund has been formed, to which those who are interested in nurses may make contributions, to be applied to the augmentation of the amount of the pensions. The extent of the influence which this bonus fund will exercise in increasing the pensions will be appreciated from the fact that about £6,000 has already been contributed to it, excluding the Chancery deposit of £20,000. It is confidently anticipated that the progress of the bonus fund will be such as to enable the governing body to grant pensions nearly double in amount those set forth in the published tables.

The council of the National Pension Fund see plainly, however, that even under these conditions the premiums chargeable are formidable sums to those whose incomes are only £20 a year. I will take the case of a nurse, aged 30, wishing to qualify for a pension of £15 a year at 60 years of age. For this the premium payable would be £4 5s. per annum (or 1s. 7½d. per week).†

Now, although it is expected that the bonus fund will increase the pension receivable upon this premium to not less than £26 a year (or 10s. per week)—which authorities agree in thinking would be the figure best adapted to the case of nurses who have nothing to look to but their own earnings—yet it is clearly not within the reach of nurses themselves to pay so considerable a sum as £4 5s. a year.

Under these circumstances the council of the fund prefer a special claim to the hospitals to help the nurses by paying a part of the premiums of all those who decide to make some provision for their old age by joining the National Pension Fund.

Before going into the particulars of their proposal, it will be well to consider for a moment its bearing upon our own hospital.

It has always been recognised by the board that it is just and desirable that a pension should be granted to nurses leaving the service of the hospital through age or infirmity after long and faithful services. Indeed, it is difficult to see how it could be otherwise, since no one, probably, would be disposed to allow an old and faithful servant, whose average wages had not exceeded £30, to spend in want the declining years of a life whose prime had been given to the service of the hospital.

Three nurses are at present in receipt of pensions, two of £30 and one of £20 a year.

The obligation, therefore, is acknowledged.

The adoption of the proposals of the National Pension Fund would enable the governors to fulfil this obligation at much less expense to the hopital, and that expense would be distributed over a series of years, instead of being concentrated, as at present.

I will now state definitely what the proposal of the National Pension Fund is.

It is that the hospitals should become affiliated to the fund by paying a portion—half is suggested—of the premiums of all nurses in their service (with certain limitations) who join the fund. Let us see how the nurse will stand affected if this proposal is adopted.

1. If, as at present situated, she joined an ordinary pension or annuity fund at the age of thirty to qualify for a pension of £15 at sixty years of age, she would have to pay an annual premium of £4 5s. In the National Pension Fund —if the council's plans are carried out in their entirety—the operation of the bonus fund on the one hand, and the payment by the hospital of half the premium on the other, will secure to her an annuity of £26 for an annual payment by her of £2 2s. 6d.

To make the comparison still more clear.

In the first case, unaided, for every £1 of pension she enters for she will have to pay in annual premium 5·6 shillings.

In the second case the same pension will cost her in annual premium 1·63 shillings.

2. If the nurse should from any cause withdraw from the fund before becoming entitled to pension, every penny she has paid in will be returned to her.

Thus, if she remains in the fund, she is sure of her pension, and if she leaves, her money is returned to her.

The claims of the scheme to adoption by the hospital appear to be mainly as follows:

1. Affiliation to the fund will enable the governors to award pensions to such of their nurses as become entitled to them at much less expense than at present, and that expense by being distributed over a longer period will be much less felt, as has been already stated.

2. If affiliation were decided upon, the hospital would, of course, definitely make it known that no nurse would in future be paid a pension, except through the Pension Fund. The decision, therefore, rests with the nurse. If she decides to make some provision for old age, the hospital will second her efforts, but if she does not, then she understands that she has nothing to anticipate in the way of pension from the hospital.

3. The scheme places in the hands of the hospital authorities a ready means of inculcating among their nurses habits of thrift and providence.

4. The hospital retains control over all our moneys paid by it to the National Pension Fund on behalf of its nurses. That is to say, if from short period of service, misconduct, or other cause, nurses should not become chargeable on the fund, the premiums paid by the hospital remain its own property as much as if they stood at its own bankers, and bear interest, moreover, at 3 per cent. The hospital, therefore, cannot suffer any loss, since it will actually make disbursements only to benefit the nurse, and if its funds do not go into the nurse's pocket they remain to the credit of the hospital and bear interest.

Note.—The council of the fund expect, however, that the hospital will decide that all sums paid by it to the fund, but not absorbed in the payment of pensions, shall be applied to the formation of a St. Mary's Nurses' Compassionate Fund, from which awards may be made to those among our nurses who, though not entitled to pension, have, from any cause, claims to consideration at the hands of the hospital authorities.

What the proposals come to, in a few words, is this:

For the nurse—that she has here the opportunity to obtain, for part payment, an article for which elsewhere she must pay the full money value.

For the hospital—that it will be enabled hereafter, if affiliated to this fund, to pay pensions to its nurses, as heretofore, but at a cost to itself of about one-third; and the pressure of that reduced charge will be minimised by being equally distributed over a term of years.

In conclusion, I would say, it seems to me, from the point of view both of the hospital and of the nurse, the proposal of the National Pension Fund is worthy of adoption; at any rate, it merits a favourable consideration at the hands of the committee. Thomas Ryan, Secretary.

St. Mary's Hospital, 1st May, 1889.

---

* I have preserved my figures, and shall be glad to produce them.

† Of course, nurses can arrange to take their pensions at 55, or any other age, or to have sick pay as well. Table F being the best one for this object, and the rates being only a little higher, or £5. 2s. per annum, of which 15s. for sick pay is not returnable.

